# Melatonin on hypothyroidism and gonadal development in rats: a review

**DOI:** 10.5935/1518-0557.20200053

**Published:** 2020

**Authors:** Yuri Mateus Lima de Albuquerque, Welma Emídio da Silva, Francisco de Assis Leite Souza, Valéria Wanderley Teixeira, Álvaro Aguiar Coelho Teixeira

**Affiliations:** 1 Departamento de Morfologia e Fisiologia Animal, Universidade Federal Rural de Pernambuco- PE, Brazil

**Keywords:** melatonin, gonads, hypothyroidism

## Abstract

We evaluated the evidence in research on the effects of melatonin on hypothyroidism and gonadal development. According to the World Health Organization, thyroid disorders due to iodine deficiency affect about 740 million people worldwide. Hypothyroidism is a thyroid dysfunction characterized by hypometabolism of the gland, with reduced or physiologically normal T3 and T4 serum levels, and high TSH level. This disorder occurs mainly in adult women in the reproductive phase, with a prevalence of 2% among the world's female population, with profound repercussions on gestation and fetal formation. During the gestational period, the thyroid is initially stimulated by high concentrations of human chorionic gonadotrophin; thus, maintaining maternal euthyroidism during pregnancy and lactation is fundamental for fetal growth and development. Besides, the hormones produced by this gland are involved in the formation of various organs, such as the skin, brain and gonads. Hypothyroidism is associated with several menstrual abnormalities, anovulation and hyperprolactinemia, resulting in a high rate of abortions, premature births, placental rupture, and weight-related neonatal deficits. In addition, there are studies showing that hypothyroidism can affect ovarian morphology (number of ovarian follicles) and testicular morphology (changes in the testicular-lumen epithelium). Melatonin is a hormone known to modulate the estrous cycle and pregnancy, and studies show that the exogenous application of melatonin increased T4 levels in female rats and controlled the decrease in T3 serum levels, reverting the sigs of hypothyroidism.

## INTRODUCTION

The World Health Organization (WHO) stated that approximately 740 million people worldwide suffer from thyroid disorders due to iodine deficiency (Abalovich *et al*., 2002). Hypothyroidism is a pathological condition of thyroid hormone deficiency that can lead to serious adverse health effects. Hypothyroidism is divided into two different biochemical types: overt, where the thyroid-stimulating hormone (TSH) concentrations are above the reference range and free thyroxine concentrations below regular range or subclinical, where although the TSH concentrations are above the reference range, free thyroxine concentrations are within the normal range (Chaker *et al*., 2017). This disorder is present in 2% of the world's female population (Hapon *et al*., 2010), interfering directly in gestation and fetal formation (Maciel & Magalhães, 2008).

A hormone that plays a regulatory role in the pregnancy and thyroid physiology is melatonin. This hormone modulates the estrous cycle and pregnancy (Maganhin *et al*., 2013) and reverses signs of hypothyroidism in hypothyroid rats with the administration of exogenous melatonin (Bondarenko *et al*., 2011).

This review summarizes the connection between melatonin with hypothyroidism and gonadal embryogenesis.

## LITERATURE REVIEW

### Thyroid

The thyroid gland weighs 20 grams on average in the human species, and 40 milligrams in rats. It comprises two lobes and an isthmus that unites them. In addition, the pyramidal lobe, which may originate from one of the lobes or from the isthmus itself, is present in 12-65% (Soukup *et al*., 2001; Ayadi *et al*., 2017; Kaklamanos *et al*., 2017). While each lobe is 4-5 cm high, 2-3 cm wide and 2-4 cm thick, the lobes are usually located between the first and fourth tracheal rings. Left and right lobes partially surround the front trachea. Laterally, there is the carotid sheath and the sternocleidomastoid muscle (Menzilcioglu *et al*., 2016).

The thyroid gland in humans develops from the neural crest and the primitive pharynx, assuming its position still attached to the thyroglossal duct. The thyroid is one of the first endocrine glands to become active in humans, being composed of thyroid follicles, formed by cubic epithelial cells, the thyrocytes (Menzilcioglu *et al*., 2016). Thyrocytes have polarity: the basal zone is related to the interstitial connective tissue, where the vessels and nerves pass, while the apical pole points to the light of the follicle. The apical zone has pseudopods that play a fundamental role in capturing the elements of the colloid, hormonal synthesis and its release (Chastain & Ganjan, 1986).

The thyroid by through the TSH stimulation (thyroid stimulating hormone) synthesizes thyroxine (T4) and triiodothyronine (T3) hormone, regulating the body's metabolism, of fundamental importance in embryogenesis (Menzilcioglu *et al*., 2016). However, unlike humans, in which thyroid hormones are secreted even in the first trimester of gestation (Smallridge & Lendenson, 2001), in rats the development of the thyroid is slower, becoming active and producing the thyroid hormones around the 17^th^ day of gestation (Choksi *et al*., 2003), making the rodents’ fetuses’ embryogenesis dependent on maternal thyroid hormones.

After birth, the thyroid and the hypothalamic-pituitary-thyroid axis in rats are immature compared to humans. During the first 21 postnatal days, this gland grows due to increased colloid deposition and follicular cell proliferation, which remains constant from birth until the 21^st^ day of life, reducing after that period (Parker & Picut, 2016). Histologically, throughout the postnatal period in rats, the thyroid follicles tend to be wider at the periphery of the gland, representing a progression in follicle maturation from the center to the periphery. Rats’ thyroids fully develop on the 21^st^ postnatal day, but becomes endocrinologically complete only on the 28^th^ postnatal day (Parker & Picut, 2016). In addition to follicular cells, the thyroid gland contains the C cells, which produce calcitonin. These cells become visible in a light microscope around the 21^st^ postnatal day, having a low mitotic activity until the 42^nd^ postnatal day. It is reported that from birth to the 120^th^ postnatal day in rats, the number of C cells increases nine fold, and their size increased by up to four times (Parker & Picut, 2016).

Its sexual dimorphism in rats becomes apparent around the 40^th^ postnatal day, in which the male gland has less colloid than in females, and its cells have cytoplasmic vacuoles (Parker & Picut, 2016). This dimorphism is due to the male follicular epithelium synthesizing the androgen hormone through the TSH feedback, where the levels of this hormone are lower in male rats until the 21^st^ postnatal day (Banu *et al*., 2002).

### Hypothyroidism

Hypothyroidism is a common thyroid dysfunction, characterized by hypometabolism of the gland, and it can be identified as overt hypothyroidism when T3 and T4 serum levels are reduced, and the TSH level is elevated (Welsh & Soldin, 2016; Feldt-Rasmussen & Klose, 2016); or as subclinical hypothyroidism, when serum levels of T3 and T4 are physiologically normal, and TSH levels are elevated (Shizuma, 2016).

Thyroid disorders represent a major public healthcare problem worldwide, following diabetes as the most common endocrine disorder in adult medical practice, and presenting a myriad of devastating consequences if not treated in advance (Vanderpump, 2011). The epidemiology and clinical features of thyroid disease are determined by the supply of iodine, an essential element in the synthesis of thyroid hormones. In addition, excessive variations in iodine levels may represent adverse health effects (Brotfain *et al*., 2013), such as increased or decreased metabolic rate and thermogenesis; and it is also correlated with an increase in body mass index (BMI) and obesity (Yim, 2016).

The relationship between TSH and free T4 is so sensitive that a small decrease in free T4 may result in an increase in serum TSH, raising its level above the reference range, while the free T4 level is still within the standard levels (Surks *et al*., 2004). Subclinical hypothyroidism is a disorder that occurs most frequently in women, the elderly and in areas where there is a greater intake of iodine. The prevalence rate varies from four to 10% in the adult population, and if there is an increased intake of iodine, it goes up to 24% (Biondi & Cooper, 2008; Vanderpump, 2011). In 80% of patients with subclinical hypothyroidism, there are a greater number of anti-thyroid antibodies, which means that in most cases an autoimmune process is present, causing such a condition (Fatourechi, 2009).

The clinical course of subclinical hypothyroidism may progress towards the development of overt hypothyroidism, as well as toward the normalization of TSH values. A study carried out with 82 women with increased TSH levels showed that after a 10-year period, 28% of them developed overt hypothyroidism; 68% of them still had subclinical disorder, while 4% of them had normal TSH (Huber *et al*., 2002).

Díez & Iglesias (2004) examined the natural course of subclinical hypothyroidism in 107 patients, and showed that patients with the mild disorder (TSH levels of 5.0 to 9.9 mU/l) are more likely to have normalized TSH values compared to patients whose TSH is greater than 10.0 mU/l. There are also reports that the TSH value was the most important prognostic factor for the diagnosis of subclinical hypothyroidism (Díez & Iglesias, 2004).

### Hypothyroidism and pregnancy

Since ovarian and placental development depend on the complex interaction between endocrine, paracrine and autocrine factors, thyroid dysfunctions compromise fetal fertility, gestation and development in humans and rodents (Choksi *et al*., 2003; Hapon *et al*., 2010).

Thyroxine (T4) and triiodothyronine (T3) act on ovarian and placental tissue, modulating their metabolism and development (Galton *et al*., 2001; James *et al*., 2007). Specific receptors for T3 are present in the nucleus of ovarian cells, so that this hormone can have a direct effect on these tissues (Evans *et al*., 1983; Maruo *et al*., 1992). However, the placenta, in addition to expressing receptors for thyroid hormones (Leonard *et al*., 2001), accumulates and metabolizes T3 and maternal T4 (Calvo *et al*., 1992). Thus, thyroid dysfunctions are associated with several ovarian and placental morpho-functional changes with impaired reproductive efficiency (Choksi *et al*., 2003).

Hypothyroidism is common in adult and reproductive women, with a prevalence of 2% in the worldwide female population (Hapon *et al.,* 2010). The incidence of hypothyroidism during pregnancy is between 0.3% and 2.5% (Idris *et al*., 2005), having profound repercussions on gestation and fetal formation (Maciel & Magalhães, 2008). During gestation, the thyroid is initially stimulated by high concentrations of human chorionic gonadotrophin (hCG); thus, maintaining maternal euthyroidism during gestation and lactation, which is critical for fetal growth and development (Maciel & Magalhães, 2008), since the hormones produced by this gland are involved in the formation of organs such as the skin (Amerion *et al*., 2013), brain (Morreale de Escobar *et al*., 2004) e testis (Wagner *et al*., 2008) of the fetuses. Hypothyroidism is associated with several menstrual abnormalities, anovulation and hyperprolactinemia (Sanyal & Raychaudhury, 2016), resulting in a high rate of miscarriages, premature births, placental rupture, and weight-related neonatal deficiencies (Amerion *et al*., 2013).

Adaptive changes in the maternal thyroid occur during gestation, in response to the need to provide the fetus with T3 and T4 until the fetal hypothalamic-pituitary-thyroid system is functional. For this, the maternal thyroid increases in volume, as well as its uptake of iodide (Versloot *et al*., 1997). In addition, estrogen levels stimulate the expression of TBG (thyroxine binding globulin) in the liver and almost double their serum concentration. The serum increase of TBG occurs concomitantly with the increase of total serum concentrations of T3 and T4 (Karabinas & Tolis, 1998).

The main function of T3 is to regulate cell carbohydrates and proteins metabolism. Thus, changes in T3 plasma levels can affect all organs and organ systems, with important effects on the cardiovascular, nervous, immune and genital systems (Choksi *et al*., 2003). T3 levels in laboratory rodents influence the control of the estrous cycle, behavior, maintenance of pregnancy, fetal growth and lactation (Vasudevan *et al*., 2002). The deiodinases, proteins responsible for the activation of thyroid hormones present in human and rodent placentas rapidly metabolize maternal T4 to T3, which will be used by the fetus, with a significant amount of T4 also being transferred (Chan & Kilby, 2000). The placenta is freely permeable to iodine and thyrotropin releasing hormone (TRH), but not to TSH. We believe that maternal TRH transferred to the fetus may play an important role in the control of fetal thyroid function before complete maturation of the hypothalamic-pituitary-thyroid axis.

The authors reported that rat gestations induced to hypothyroidism is prolonged, lasting about 24 days, and generating on average nine pups per gestation, number smaller than that of the control group average, which are usually of 12 pups. In addition, rats with low levels of thyroid hormones exhibit higher levels of progesterone at the end of gestation, as well as lower levels of estrogen and litters with lower weights when compared to data from control groups (Hapon *et al*., 2003). Another observed effect relating this dysfunction to gestation is the prolongation of corpus luteum function in pregnant rats (Hapon *et al.,* 2007), where there is a reduction in the proliferation, apoptosis and expression of angiogenic factors in the corpus luteum of pregnant rats (Silva *et al*., 2014).

Induced hypothyroidism compromises the placental layers of the rat, and it increases glycogen cell population in the spongiotrophoblast layer relative to the cytotrophoblastic cells, and interfere with the vascular development of the placental labyrinth, thus reducing proliferative activity and cellularity, and increasing the apoptotic rate of the three layers of the placental disc (Silva *et al*., 2012). This morphological alteration caused by such dysfunction may also cause low body weight of the litters of rats induced to hypothyroidism, this is because there is a reduction in the area occupied by fetal capillary in the placental labyrinth at 14 days of gestation, and may be insufficient to establish with maternal blood, causing low fetal weight (Silva *et al*., 2012).

Thus, a state of clinical or subclinical hypothyroidism may be worsened by the pregnancy state, and adequate function of the mammary glands may be impaired. The impact on mother and offspring is well documented, and one of its most pronounced consequences is delayed growth and delayed maturation of the newborn, causing mental retardation and subnormal height. Although most of these effects are attributed to the hypothyroid state of infants, any change in maternal metabolism that could lead to decreased milk production or excretion could further complicate offspring development (Hapon *et al*., 2003).

### Gonadal embryology

Gonad development has two phases. The initial phase has the appearance of the so-called indifferent, bipotential gonad or genital crest, which is identical in males and females. The cell lines that compose it are bipotential, being able to turn into male or female gonads. The second stage is testicle or ovary development (Wilhelm *et al*., 2007). The gonads develop from the intermediate mesoderm, which is situated in the longitudinal urogenital crests; the most medial part of these crests are the gonadal ridges, occurring during the fifth week of gestation in humans, and around the 10^th^ day of gestation in rats (Zayed *et al*., 2007). During development, the primordial germ cells migrate from the yolk sac wall through the dorsal mesentery of the large intestine to occupy the gonadal ridges. The arrival of these cells induces the cells in the crests to form primitive sex chords (derived from mesonephros and overlapping celomic epithelium). At this stage, the gonad is indifferent to sex, and consists of an external cortex and an inner medulla. If the germ cells do not migrate to the gonadal ridges, the gonads do not grow (Mitchell & Sharma, 2009).

In embryos with no testicle-determining factors, the primitive sexual cords extend into the gonad medulla, degenerate and form a vascular stroma, resulting in an ovary (Mitchell & Sharma, 2009). Regarding ovarian development, in the postnatal day (PND) 3, primordial, primary and secondary follicles are apparent, with a thick peripheral cortex of primordial follicles and a central nucleus of primary and secondary follicles. At no other time in the development of the ovary is the primordial follicle the most prominent feature. In PND 10, the secondary and early antral follicles are immature compared to the follicles of the adult animal in the active estrous cycle. Around PND 21, large antral follicles can be seen occasionally within the medulla. These large antral follicles in the medulla of the infant ovary can be distinguished from the ovulatory follicles of the peripuberal and mature ovary, since the ovulatory follicles that eventually ovulate are more commonly located in the external ovarian cortex. In PND 30, the most apparent morphological characteristic is the appearance of the ovulatory follicle in the external cortex. These follicles are of sufficient size for ovulation and have a distended antrum and a primary oocyte coated by the cumulus oophorus layer (Picut *et al*., 2014; 2015a).

In embryos with testes determining factors, the primitive sexual cords proliferate and penetrate the medulla forming the testicular cords. Some of these cells differentiate into Sertoli cells, while the remaining form the seminiferous tubules. The testicular cords anastomose to form the rete testis, which becomes continuous with 15 to 20 persistent mesonephric tubules, the efferent duct. The testes determining factor also induces the differentiation of mesenchymal gonadal cells into interstitial Leydig cells (Mitchell & Sharma, 2009). We believe that Sertoli cells act as the organizing center of the male gonad, and orchestrate the differentiation of all other cell types (Wilhelm *et al*., 2007).

In PND 3, the testes consist of gonocyte-coated tubules and mitotically active Sertoli cells. In PND 15, spermatogonia are still mitotically active and spermatogonia reach the maximum density forming a thick pseudo-stratified layer with Sertoli cells. The mitotic rate in the population of spermatogonial cells decreases compared to that of the early childhood period (PND 3), and apoptotic spermatogonia are present in the center of the tubules. The period between the PNDs 21 to 30 holds the maintenance of the first wave of spermatogenesis in rounded spermatids, and mainly by a significant increase in the tubular diameter (Picut *et al*., 2015b).

### Melatonin

The pineal gland produces and secretes melatonin and other peptides still poorly defined, through the release of noradrenaline (NE) by intraparenchymal nerve fibers, where this release and activity of the pineal gland are activated in dark environment and inhibited by light (Maganhin *et al*., 2009; Junqueira & Carneiro, 2013). Also known as N-acetyl-5-methoxytryptamine, melatonin derives from the serotonin that has tryptophan as the precursor, and is the main product of the pineal, exhibiting high solubility and a light yellow stain. It is transported through the plasma connected to proteins such as albumin (Sumaya *et al*., 2005; Maganhin *et al*., 2008).

With specific receptors in cell membranes (MT1 and MT2), melatonin has several functions as modulating the circadian cycle of antioxidant enzymes, bone metabolism, growth of ovarian follicles, ovulation, luteinizing hormone, fertilization and implantation (Tamura *et al*., 2009; 2014; Sharma *et al*., 2015). This hormone can also exert antioxidant functions, due to its small molecular size and its lipolipid properties, being able to cross all cell membranes and reach intracellular compartments, as well as the nucleus and mitochondria, organelles with high concentrations (León *et al*., 2005; Waseem *et al*., 2017), preventing damage to DNA (Sousa Coelho *et al*., 2018). The reduction in the occurrence and growth of tumors causes melatonin to be the most important natural oncostatic hormone in the human body (Reiter, 2004; Cabrera *et al*., 2010). We also believe that melatonin plays an important role during the life cycle, acting in growth, development, maturation and aging, decreasing its plasma concentration with the individual's age (Tamura *et al*., 2009; 2014).

Melatonin is cited in several studies as an important part of the neuroendocrine system, influencing the control of circadian rhythms and controlling various physiological processes (Ferreira *et al*., 2010). The sites in which the interaction between melatonin and the endocrine system occur are still unclear. We believe that the activity of this hormone involves the hypothalamus, the pituitary, the gonads, and the pineal gland, the main one responsible for its production (Reiter, 1995).

### Melatonin and the gonads

Melatonin is a broad-spectrum functional hormone responsible for regulating internal hormonal changes in response to variations according to periods of light and darkness (Mukherjee & Haldar, 2014). In photoperiodic species, melatonin secretion by the pineal gland is responsible for the effects of day length on the seasonal reproduction cycle (Maldonado *et al*., 2012). In these species, melatonin has a pro-gonadotrophic effect, increasing follicle-stimulating hormone (FSH) and luteinizing hormone (LH) peak concentrations, probably by blocking the inhibitory effects of sex steroids on ovulation (Rocha *et al*., 2011).

In species with non-seasonal reproduction, that is, non-photoperiodic, pre-ovulatory release of gonadotrophins is controlled by a circadian cycle. These species present a daily circadian rhythm of melatonin release (Claustrat *et al*., 2005). There are melatonin receptors, MT1 and MT2, in both male and female gonads of these species (Maganhin *et al*., 2008; Mukherjee & Haldar, 2014), thus reaffirming its antigonadotrophic properties, such as inhibition of gonadal development, spermatogenesis and androgen production in males and absence of follicles, corpus luteum and proliferation of interstitial tissue in females (Soares Jr *et al*., 2003).

Melatonin plays a significant role in fetal programming, since the follicular fluid has high concentrations of it, suggesting its direct participation in oocyte maturation and embryo development, due to its ability to reduce oxidative stress in the ovarian follicles and to protect these oocytes of free radical damage (Nelissen *et al*., 2011; Brzezinski *et al*., 1987). Melatonin levels in maternal plasma may increase during pregnancy, however, in compromised pregnancies, this hormone in the mother and fetus may be affected (Chen *et al*., 2013).

As for female gonads, the mechanisms that control folliculogenesis are still unclear; however, hormones and several growth factors are involved (Escames *et al*., 2012). Studies have demonstrated the presence of melatonin receptors (MT1 and MT2) in the ovarian follicles, thus supporting the hypothesis of its performance in ovarian physiology (Soares Jr *et al*., 2003; Lee *et al*., 2014). Regarding the male gonads, melatonin plays a protective role in testicular development both *in vitro* and *in vivo*, as well as regulates it by controlling the secretion of neurohormones (particularly GnRH) and testosterone (Li & Zhou, 2015).

According to Gholami *et al*. (2013), melatonin may improve the structure of testicular tissue. Such researchers have observed that this hormone can induce cell proliferation in normal cells and induce apoptosis in damaged cells (Gholami *et al*., 2014) (Niu *et al*. 2016); however, its addition to the spermatogenic stem cell (SSCs) culture medium could increase SSC proliferation by stimulating glial-derived neutrophilic factor (GDNF) production in the Sertoli cells. Furthermore, low levels of melatonin during pregnancy and lactation matrix results in an involution of the testes offspring, indicating that their levels during pre and postnatal development interfere with testicular growth (Tuthill *et al*., 2005). We also know that pinealectomy increases testicular weight, while administration of exogenous melatonin decreases the above-mentioned weight in non-pinealectomized rats (Kuş *et al*., 2000; Akosman *et al*., 2013).

### Melatonin and thyroid

There is a significant relationship between the thyroid and pineal glands, suggesting that deficiencies in thyroid function may alter the release of melatonin (Rom-Bugoslavskaia & Bondarenko, 1984). Hypothyroidism causes a significant decrease in plasma melatonin levels, when we compare rodents induced to it to healthy rodents. Rats induced to hyperthyroidism have higher levels of this indolamine in plasma, suggesting a relationship between the thyroid disorders and the pineal gland (Belviranli & Baltaci, 2008; Baltaci & Mogulkoc, 2017; 2018). Likewise, Bondarenko *et al*. (2011) demonstrated that signs of hypothyroidism in rats with low levels of melatonin due to exposure to constant light were reversed with their exogenous application.

Laskar *et al.* (2015) investigated the presence of receptors of this hormone in the thyroid gland, and reported that exogenous application of melatonin increased T4 levels in female rats. Their results were also found by Skipor *et al*. (2010) that, through the exogenous application of this indolamine, found a prevention in the decay of serum T3 levels and a control in the decay of T4 levels.

### Effects of hypothyroidism on morphometry and cell proliferation

There are several studies showing that thyroid hormones affect spermatogenesis by promoting changes in basal metabolic activity and cellular respiration of the testes (Oppenheimer *et al*., 1987; Mutvei & Nelson, 1989; Fadlalla *et al*., 2017), or by affecting Leydig cells, resulting in the reduction of testosterone secretion (Zirkin *et al*., 1980; Mendis-Handagama *et al*., 1991). In addition, in hypothyroid rats, testicular morphology is also affected, where there is a modification in the relation of the testicular-lumen epithelium, causing changes in lumen size (Ai *et al*., 2007).

Previous studies have shown that such a thyroid disorder can also affect ovarian morphology by altering the number of ovarian follicles (Meng *et al*., 2017). The transition from the primary to the secondary follicle (pre-antral stage) is controlled by intra-ovarian factors such as GDF-9 (Differentiation Factor and Growth-9) (McGrath *et al*., 1995; Orisaka *et al*., 2006). We know that the thyroid influences this mitogenic factor, although the mechanism is still not fully understood (Dong *et al*., 1996; Hayashi *et al*., 1999).

Along with morphological changes, we know that during pregnancy, in humans and rodents, the uterus undergoes a series of morphofunctional changes in order to accommodate the growing embryo, but the hypothyroid state decreases the proliferative rate of epithelial cells, stroma and myometrium by reducing the response of uterine cells to estrogen (Kirkland *et al*., 1981). In addition to this organ, hypothyroidism may also cause a decrease in the expression of proliferative antigens in placentas and testes of rats (Silva *et al*., 2012; Fadlalla *et al*., 2017), thus showing the anti-proliferative effect of hypothyroidism.

## CONCLUSION

This selective literature review supports the proposition that maternal hypothyroidism affects the development of the embryo, focusing specially on their gonads. As it is apparent from the studies hereby mentioned, melatonin may play a role in the protection of the effects of hypothyroidism in both mothers and their offspring, by preventing the decrease in thyroid hormone levels in rats, and reversing signs of hypothyroidism in rats. Additionally, melatonin seems to interfere directly in the embryogenesis of both gonads, since there are receptors for this hormone in ovaries and testicles. In addition, it is still unclear how melatonin affects the ovaries. We know that in testicles, melatonin plays a protective role by stimulating normal cell proliferation, but it induces apoptosis on damaged cells. We hope that because of this paper the interest in the effects of hypothyroidism in the embryology of fetuses from hypothyroid mothers will increase, giving relevance to a disease that affects a growing number of females in reproductive ages, since studies on this topic are scarce.
